# Identification of Bicluster Regions in a Binary Matrix and Its Applications

**DOI:** 10.1371/journal.pone.0071680

**Published:** 2013-08-05

**Authors:** Hung-Chia Chen, Wen Zou, Yin-Jing Tien, James J. Chen

**Affiliations:** 1 Division of Bioinformatics and Biostatistics, National Center for Toxicological Research, U.S. Food and Drug Administration, Jefferson, Arkansas, United States of America; 2 Graduate Institute of Biostatistics and Biostatistics Center, China Medical University, Taichung, Taiwan; 3 Institute of Statistical Science, Academia Sinica, Taipei, Taiwan; National Taiwan University, Taiwan

## Abstract

Biclustering has emerged as an important approach to the analysis of large-scale datasets. A biclustering technique identifies a subset of rows that exhibit similar patterns on a subset of columns in a data matrix. Many biclustering methods have been proposed, and most, if not all, algorithms are developed to detect regions of “coherence” patterns. These methods perform unsatisfactorily if the purpose is to identify biclusters of a constant level. This paper presents a two-step biclustering method to identify constant level biclusters for binary or quantitative data. This algorithm identifies the maximal dimensional submatrix such that the proportion of non-signals is less than a pre-specified tolerance δ. The proposed method has much higher sensitivity and slightly lower specificity than several prominent biclustering methods from the analysis of two synthetic datasets. It was further compared with the Bimax method for two real datasets. The proposed method was shown to perform the most robust in terms of sensitivity, number of biclusters and number of serotype-specific biclusters identified. However, dichotomization using different signal level thresholds usually leads to different sets of biclusters; this also occurs in the present analysis.

## Introduction

Recent advances in biotechnology have generated massive amounts of data to understand biological processes, discover new targets and new drugs, predict toxic potential of unknown compounds, or identify pathogens in outbreak source tracking. A dataset can be expressed in a two-way table with rows representing the measured attributes and columns representing samples. Cluster analysis is a commonly used data mining technique to explore the relationships among attributes and among samples and to identify patterns and structures between the attribute and sample relationships. Cluster analysis and data mining of binary data matric also arise in many scientific applications, such as document-term data in bioinformatics, species characteristics in systematic biology, and genotyping and gene expression data in genomics. For example, in document clustering, each document can be represented as a binary vector where each element indicates whether a given word/term was present or not [1]. In gene expression data, the intensity levels were converted to binary data to detect transcriptional change of *Saccharomyces cerevisiae* under various environmental conditions. The binary values are noisy indicators of the presence or absence of mRNA in a *Saccharomyces cerevisiae* cell [2].

Clustering techniques provide a global analysis of samples by grouping samples with similar attributes in the same cluster, and samples with dissimilar attributes are in different clusters or vice versa. However, cluster analysis does not provide information for understanding local relationships between samples and attributes. In many applications, discovery of a subset of attributes that are associated with a subset of samples is of primary concern. In gene expression experiments, functionally related genes may exhibit a similar pattern in only a subset of samples, not in all samples. An interest of the study is to identify those co-expressed or co-regulated genes that are associated with the certain subsets of samples. A biological indication of those co-regulated genes is that they may play similar functional roles in cells due to their closely correlated expression patterns. In an un-weighted network analysis methodology focuses on genes with high correlations and only the directions of expression changes (up or down) are considered in the analysis instead of the magnitudes [3], [4]. Identification of these genes helps in searching for their upstream transcriptional regulator associated with experimental conditions. In pharmacovigilance, the Adverse Event Reporting System (AERS) database, which consists of over 8,000 drugs and over 10,000 adverse events reported, is the primary database designed to support the FDA's post-marketing safety surveillance program for all approved drugs and therapeutic biologic products. The goal is to identify which sets of drugs are associated with which sets of adverse events. Because of the frequency of reports is not necessarily informative regarding the number of individuals taking the drug, a pre-determined threshold cutoff is used to dichotomize the signals and noises [5]–[7]. In food safety surveillance, serotyping of isolates are used for identification and characterization of *Salmonella* isolates in outbreak investigations. The Pulsed-field Gel Electrophoresis (PFGE) has been used as the “golden standard” to confirm an outbreak of a disease and determine its possible source [8]. In PFGE analysis, the fingerprint of an isolate is characterized by the presence or absence at designated band locations. A goal is to identify the subset of band locations that can characterize the serotype of outbreak isolates.

Biclustering has been developed to identify submatrices in a two-way data matrix in which rows and columns are correlated [9]–[28]. Biclustering techniques identify subsets of attributes that exhibit “coherence” patterns with a subset of samples. The data matrix consists of a collection of submatrices (biclusters), each representing an association between a set of attributes and the corresponding set of samples. Many biclustering methods have been proposed, each method was developed to subject a specific mathematical formulation and focus to identify specific bicluster patterns. Most if not all bicluster algorithms were developed to detect regions of “coherence” patterns. However, the “coherence” of a bicluster can be defined in several ways, and most of them are defined as a submatrix having additive correlation on column, row, or both of them. Madeira and Oliveira [29] discerned four different categories of biclusters: 1) constant biclusters, biclusters with a constant value, 2) biclusters with constant values on either columns or rows, 3) biclusters with coherent values, and 4) finally biclusters with coherent evolutions. Therefore, it cannot be expected that a single method is well suited for all scenarios. A recent comparative study showed that there were significant differences in performance among biclustering methods, depending on the problem that was examined [16]. The BicAT toolbox [17], freely available at http://www.tik.ee.ethz.ch/sop/bicat, provided several biclustering algorithms for different biological applications [29]–[32].

Prelic et al. [16] proposed Bimax for bicluster analysis of binary data (identifying constant level biclusters). For the analysis of gene expression data, the model assumes two possible states in the data, a gene is either “on” (change- signal) or “off” (no change- non signal) with respect to a control or reference condition. The Bimax method used the midrange to dichotomize gene expression data into signal and non-signal groups. Genes showing no change, including those in the background region, would be classified as non-signals. This modeling approach will reduce the number of false identifications considerably. Bimax was shown to have the best performance compared to five other prominent biclustering algorithms. Alternatively, Kluger et al. [21] proposed the Spectral method using the singular value decomposition (SVD) approach, and Carmona-Saez et al. [22] proposed the nsNMF (non-smooth non-negative matrix factorization) method for biclustering of gene expression data. Neither method requires specifying the number of biclusters, but rather requires pre-specifying the rank of factors to represent the data matrix. The SVD and NMF methods identify local regions where the attributes and samples are correlated and do not target specific patterns of correlation. Recently, Zhang et al. [33] extended the NMF to binary matrix factorization (BMF). Like Bimax, this approach requires dichotomizing the gene expression data matrix into binary matrix. The biclustering results from NMF and BMF may not be reproducible in different runs. The use of the SVD or NMF for bicluster analysis will be referred to as the matrix factorization approach. Rodriguez-Baena et al. [34] proposed the BiBit algorithm, based on a fast bit-pattern processing technique; BiBit performed similar to Bimax, but used significantly less computation time.

In a binary matrix, ‘1’ and ‘0’ encode the presence and absence of a signal, respectively. The number of 0 s (non-signals) is often much larger than the number of 1 s (signals). For example, the proportion of the signal in the AERS data is less than 0.10. In gene expression data matrix, many genes, including the background intensities, exhibit no difference in expression among samples. The interest is to find the biclusters that contain (almost) one. The use of methods that are developed to detect regions of coherence patterns to identify constant biclusters in a binary matrix can be inefficient, as compared to the biclusters algorithms that are developed for binary matrix such as Bimax as discussed above. However, the Bimax often identifies many small biclusters when the proportional of signal is very low. (More discussions are given in the next section.) Furthermore, many biclustering algorithms require specification of several parameters such as random seed, minimal numbers of column and row, number of clusters, tolerance threshold in the cluster, etc. Different initial specifications may produce different bicluster results; therefore, the interpretation of the bicluster results often requires additional post-analysis techniques for validation and visualization.

Motivated by these issues, this paper proposes a two-step method for bicluster analysis of quantitative and binary data. The proposed method only requires pre-specified two parameters: 1) a threshold cutoff to dichotomize signals and non-signals for analyzing quantitative data, and 2) a threshold proportion to allow some non-signal data in the biclusters. The first step uses matrix factorization techniques to uncover the bicluster structures in the data matrix. The second step presents an edging algorithm to determine the bicluster regions. The edging algorithm is developed for binary data. The quantitative data can be dichotomized before or after the matrix factorization. The proposed methods are evaluated and compared with other biclustering methods for two synthetic and two real datasets.

## Methods

This section reviews several biclustering methods considered in this paper. Biclustering methods can be categorized as the three groups: model-based, categorical state, and matrix-factorization algorithms. The model-based methods assume specific bicluster structures and use some optimization methods to identify the biclusters. The categorical state methods categorize data into different states and develop algorithms to identify the biclusters having the same state. The matrix-factorization methods identify biclusters from the ordered data matrix according to the column and row vectors in the factorized matrices. Furthermore, we develop two matrix factorization algorithms coupled with an edging algorithm to identify biclusters and present a statistical test for significance of binary biclusters based on the Bernoulli model in [35].

### Model-Based Biclustering Methods

Most of the model-based biclustering methods assume some patterns of linear correlation between columns and rows in the bicluster, i.e. the bicluster can be modeled as the sum of row, column, and background effects, which can be estimated by row means, column means, and overall mean, respectively. Cheng and Church [18] first proposed several greedy row/column deletion/addition algorithms to identify the biclusters having smaller mean square residuals than the tolerant error δ. If the elements of data matrix *X* are *X_ij_*, the mean square residual (MSR) for a bicluster (I, J) can be obtained by
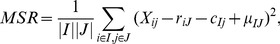
(1)where 
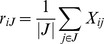
, 
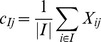
, and 
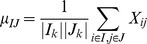
.

Lazzeroni and Owen [25] assumed the data having different structural layers and proposed an approach to identify similar biclusters which are in the different layers of the model. The elements in k-th layer were equal to the sum of the row (*r_ik_*), column (*c_jk_*), overall (*μ_k_*), and background (*μ_k_*) effects, and the data were modeled as
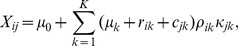
(2)where *ρ_ik_* and *κ_jk_* were the indicator functions for the membership of biclusters. They developed an iterative approach with each cycle updating the parameters to minimize the sum of square errors to identify the biclusters. Mirkin, Arabie and Hubert [26] studied a model assuming that *r_ik_*, *c_jk_*, and *μ_0_* were 0 and *μ_k_* was the maximum value in the bicluster, and developed biclustering algorithms based on difference of the sums of squared-residuals between original submatrix and itself added with a single row/column. The biclusters identified by their algorithms have the elements close to *μ_k_*'s, and the biclusters can be adjusted by the criterion for sum of squared residuals.

In addition to modeling linear correlations, product correlations in bicluster were also studied [27], and the data were expressed as
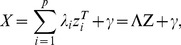
(3)where Λ, Z, and γ were sparse matrix of prototype, sparse matrix of factor, and additive noise. It assumed that the sparse matrices were distributed as *Laplace* distributions, and the noise was normally distributed. The EM algorithm was applied to estimate the parameters to identify the biclusters. The constant biclusters can be expressed as additive models having no column and row effects, that is *r_ik_* = 0 and *c_jk_* = 0 in equation (2). Alternatively, they can be described as multiplicative models having constant multipliers on row and column, that is 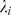 and 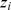 are constants in equation (3). Although some extended versions [19], [20] from Cheng and Church method [18] outperform the several existing novel methods in some scenario, these methods would not perform well on the large sparse binary matrix where the number of signals is small. In a binary matrix, unlike the quantitative data, there are only two values, 0 and 1. The 0 s can be viewed as background or noise. The model-based biclustering methods would identify many non-signal biclusters when the number of non-signals is large.

### Categorical State Methods

In genomic studies, the genes can be characterized as categorical responses, such as up-regulation, down-regulation, or unchanged (Bimax [16], BiBit [34], and xMotif [24]). In xMotif, the data were categorized into several statistically significant states, and an iterative search method using different random seeds was proposed to identify the biclusters. Bimax was developed for binary data, and the data matrix was first dichotomized as a binary matrix. A divide-and-conquer algorithm was developed to identify the biclusters in which the elements were all signals. This method performs well when there are much more signals than non-signals in the data matrix. The primary assumption for these methods is that the observed data are without random variations and errors. These methods required that each bicluster consists of only the data of the same categorization. Therefore, for the binary data, the methods do not allow any non-signal data in the biclusters. When there are non-signal data in a large bicluster, these methods will identify several small biclusters which are subsets of the large bicluster.

### Matrix-Factorization Methods

The SVD and NMF matrix factorization methods have also been proposed to identify the biclusters. The Spectral method [21] was a SVD-based algorithm; it applied the k-means technique to detect the change points to identify the biclusters. Carmona-Saez et al. [22] applied nsNMF, a variant of NMF, to search the biclusters.

**Figure 1:**
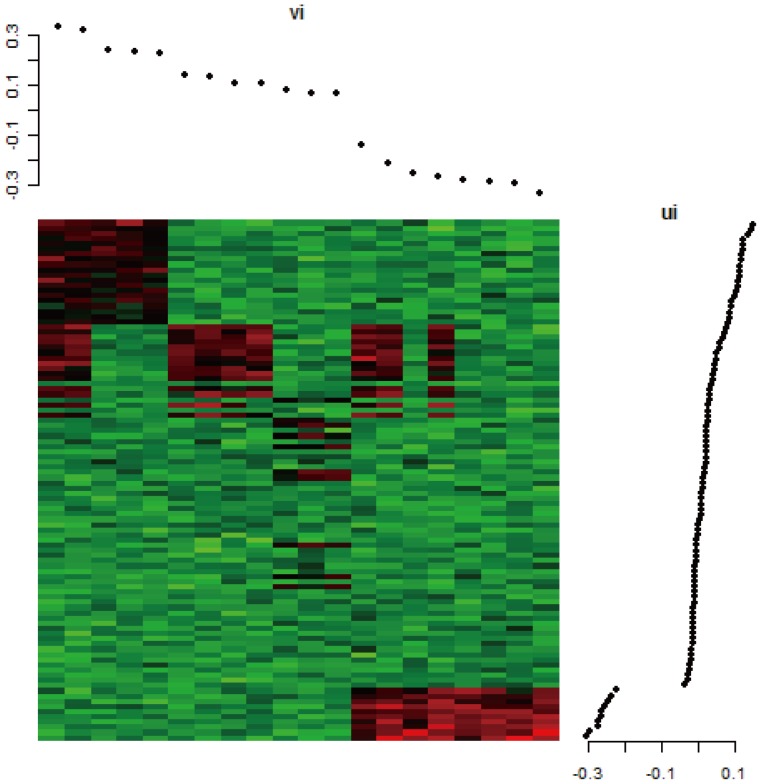
A synthetic data matrix ordered according to magnitudes of the singular vectors of second principal component. The two figures above and to the right of the ordered data matrix are the plots of the corresponding values of the singular vectors.

**Figure 2:**
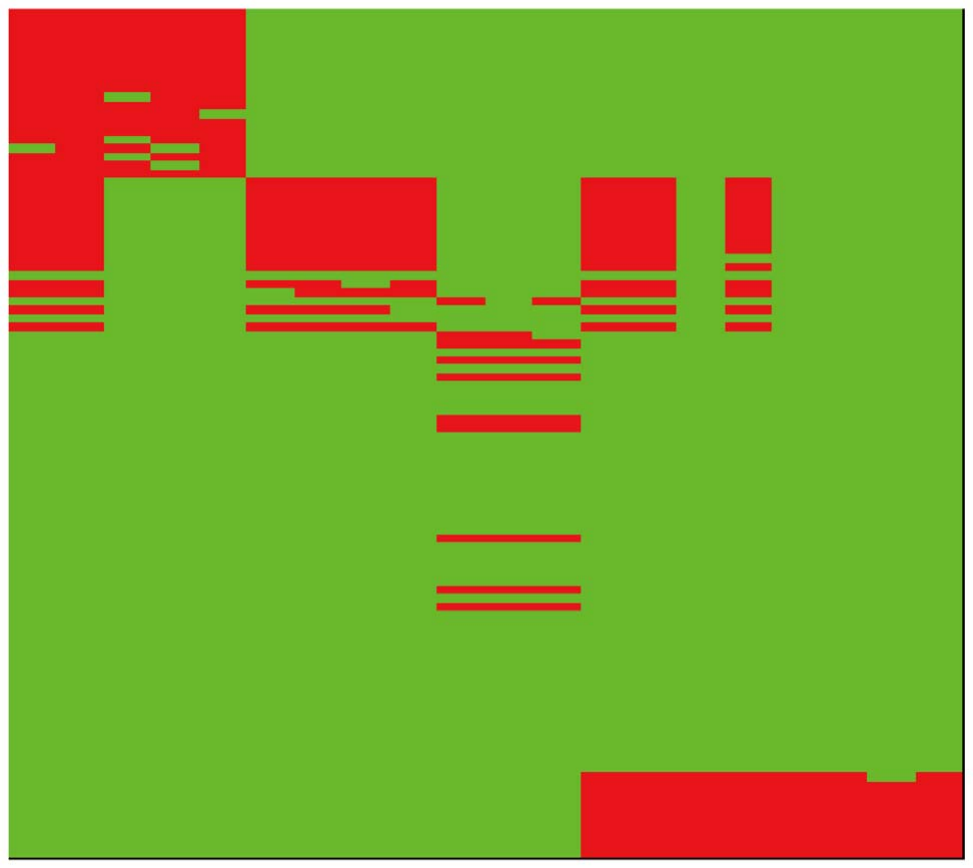
The plot of the same synthetic data matrix after data are dichotomized. The boundaries between the signals (red) and non-signals (green) are apparent.

One important issue, which was not fully addressed in the use of matrix factorization approach, is that it requires a companion segmentation algorithm to separate the signal and non-signal regions. Segmentation algorithms have been proposed to build up a bicluster region [21]–[23] by identifying row and column change points. These algorithms can fail to identify some biclusters occurred in the overlapped regions, in which there may not have a change point in the singular vectors. Figure 1 shows a data matrix where rows and columns are ordered according to the magnitudes of the singular vectors of the second principal component; the two plots shown above and to the right of the ordered data matrix are the corresponding values of the singular vectors. In these two plots, the row (right) and column (above) each has a clear change point, and therefore the bicluster structure in the lower right corner can easily be identified. However, the bicluster structure in the upper left may not be identified because the row change point may not easily be identified. Figure 2 is a plot of the same matrix after the data have been dichotomized using the two-means clustering algorithm. The boundaries between the signal and noise regions in Figure 2 are much more apparent than those in Figure 1. Furthermore, most of the aforementioned methods use different types of greedy algorithm which usually results in different bicluster sets when different random seeds are used; in addition, these methods require specification of several parameters such as minimal size, number of biclusters, and criterion for goodness-of-fit/badness-of-fit. These methods do not perform satisfactorily in our study especially for the synthetic data of simple structure (details in Result section). Therefore, we propose an alternative biclustering method based on the matrix factorization described below.

Let X denote the *p*×*n* data matrix (assuming *n* is smaller than *p* for simplicity). In SVD, the matrix X is expressed as X = ADB^T^, where A is the *p*×*n* orthonormal column matrix; D the *n*×*n* diagonal matrix, and B the n×n orthonormal matrix. The columns in A and B are eigenvectors of the matrices XX^T^ and X^T^X, and called the left singular vectors and right singular vectors. The diagonal entries (*λ_i_*) of D are the square roots of the eigenvalues of XX^T^. Writing **a** *_i_* and **b** *_i_* for *i*-th left and right eigenvectors respectively, and *λ_i_* for *i*-th eigenvalue, the SVD can be also written as
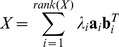
(4)in [36] where *rank(X)* is the rank of X. The first *f*<*rank(X)* terms of these summations provide a bilinear approximation for the matrix X≈RC, where R has size *p*×*f* with each of the *f* columns representing a sample basis, and C has size *f*×*n* with each of the *f* rows representing an attribute basis. The valued of *f* can be obtained from a specified proportion of the explained variance, which is the ratio of the sum of *f* largest eigenvalues to the sum of all eigenvalues. We set it to be minimum value of p and n. The columns in R and rows in C can be defined as the principal components on sample and attribute respectively, and these principal components can be derived from SVD such as R = A*_p×f_*D*_f×f_* ^1/2^ and C = D*_f×f_* ^1/2^B*_f×n_* ^T^. In NMF, the X, R and C are constrained to be non-negative matrices, and the matrix can be estimated by the objective function based on Poisson likelihood [22].

The element *x_ij_* of X can also be expressed as
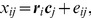
(5)where ***r*** *_i_* is the *i*-th row vector of R corresponding to the *f* sample bases, ***c*** *_j_* is the *j*-th column of C corresponding to the *f* attribute bases, and *e_ij_* is a residual. In this representation, for a given *l*-th component, the rows can be ordered by *r_il_* (the *l*-th value of ***r***_i_), and the columns by *c_lj_* (the *l*-th value of ***c*** *_j_*). The ordered X is denoted as X*_l_* (*l* = 1,.., *f*). That is, the matrix X*_l_* is ordered such that the high and low values are likely to appear in the corners. For a simple case with one bicluster having a larger constant value, a column in R and a row in C denoted as ***r*** *_.l_* and ***c*** *_l._* can well explain X such that ***r*** *_.l_* ***c*** *_l_*_._ has a minimum square error to approximate X. If SVD is applied, *λ*_1_ **a**_1_ **b**_1_ *^T^* will sufficiently approximate X. The larger values in X will be more likely to appear in the upper-left and lower-right corners of the matrix X ordered according to the values of ***r*** *_.l_* and ***c*** *_l._*. Similarly, if there are several biclusters, these patterns also can be found in some ordered X according to the different ***r*** *_.l_* and ***c*** *_l._* on row and column. In SVD, ***r*** *_.l_* and ***c*** *_l._* can be *p* **a** *_l_* and *q* **b** *_l_* where *pq* = *λ_l_* but the constant *p* and *q* do not affect the order, i.e. we only need to order X according to **a** *_l_* and **b** *_l_*. For example, for a data matrix
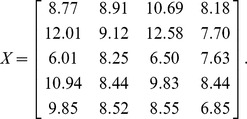



The SVD of X is given by
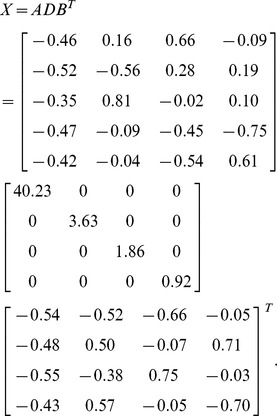



The ordered data matrix is
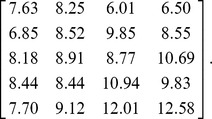



It can be seen that the high values are located in the lower-right corner. In gene expression data, the over-expressed regions will be in the upper-left or lower-right corners. The next step is to determine the bicluster regions for each X*_l_* (*l* = 1,.., *f*) which is dichotomized as a binary matrix B*_l_* using a cutoff threshold *h*, which can be estimated by the 2-means clustering algorithm or midrange. An edging algorithm to construct biclusters for binary data is described below.

Assume each element in a binary data matrix B*_l_* represents the presence or absence of a signal. Similar to the model based method of Cheng and Church [18], the proposed approach to constructing biclusters requires pre-specifying a tolerance threshold δ. A δ-bicluster is defined as a maximal dimensional submatrix such that the proportion of the non-signals in the bicluster is less than δ. That is, a δ-bicluster is a maximal submatrix covering at least (1-δ) proportion of signals, given minimum numbers of rows and columns. In other words, a δ-bicluster is a (*r*×*c*) submatrix where the proportion of 0 is less than δ and it is not a proper subset (submatrix) of any other δ-bicluster. A δ-bicluster in this paper is set at least a (2×2) submatrix. The algorithm for finding δ-biclusters is described as follows.

Segmentation algorithm to determine bicluster structure in upper-left corner of B*_l_*:

Algorithm: Segmentation

**Input**: A *p×n* binary matrix *B_l_*

**Output**: A bicluster *ul*

01: *ul*←2×2 submatrix in the upper-left corner of *B_l_*

02: **while** (number of row for *ul*) <*p* and (number of column for *ul*) <*n*

03: *pc*← proportion of 1 in adjacent column to *ul*

04: *pr*← proportion of 1 in adjacent row to *ul*

05: **if** *pc*>0.5 or *pr*>0.5

06:  **if** *pc*>*pr*

07:   *ul* merge adjacent column

08:  **else**

09:   *ul* merge adjacent row

10:  **end if**

11: **else**

12:  **break**

13: **end if**

14: **end while**

15: *q*←proportion of 0 in *ul*

16: **if** q>δ

17: *ul* is empty

18: **end if**

19: **return** *ul*

The possible bicluster located in lower-right corner of B*_l_* can be obtained by the same algorithm using reversely reordered B*_l_* on row and column. Note that the emerging submatrix after Step 14 could be expandable even if q>δ. However, this submatrix will be rejected after completion of merging in Step 18 if q>δ. An alternative approach is to check the proportion of non-signals after each step of merging. However, this alternative approach could lead to early stop merging and the identified biclusters could be smaller than true biclusters, especially for the starting 2×2 submatrices, even if there is only one non-signal, q = 0.25 and δ<0.25. On the other hand, the proposed approach of checking proportion of non-signals after completion of the merging would result in larger biclusters (step 18).

After completion of the segmentation, a collection of submatrices of candidate δ-biclusters is obtained. Each submatrix is then evaluated and compared with other submatrices. If a submatrix is a subset of another submatrix, then this submatrix will be deleted, and if two submatrices have the same rows or the same columns, they will be merged row-wise or column-wise. The final collection of matrices is the set of δ-biclusters. The segmentation algorithm has the time complexity of O(min(n, p)(n+p)). In conjunction with time complexity of SVD O(min{pn^2^,p^2^n}), the overall time complexity of the proposed algorithm is O(min{pn^2^,p^2^n}). Obviously, the computational time complexity is dominated by the SVD algorithm, and it could have high computational cost if *p* is large.

The choice of δ will affect the number and the size of the constructed biclusters; a larger δ results in more and larger biclusters. The biclusters constructed using a smaller δ will be a subset of biclusters using a larger δ. If there is no prior knowledge about the number of biclusters, a larger δ value can be specified to identify more biclusters. In summary, δ is the maximum proportion of the non-signals allowed in the bicluster, and it represents the quality of biclusters constructed; a value between 0 and 0.3 is suggested.

Non-negative matrix factorization (NMF) is an alternative approach to uncovering bicluster structures [22]. Both SVD and NMF require specification of the number of factorization components (ranks) *f*. It should be noted that the matrix factorization algorithms, for example, the SVD-based algorithms [21], [23] or NMF-based algorithms [22], [33], [37], are not designed to identify all biclusters; the algorithms can identify at most 2*f* biclusters because only the two diagonal corners in each ordered X are screened. For SVD, *f* is set to be *n*, the rank of the data matrix X. The method to estimate *f* for NMF was discussed in [22]. The edging method as described is developed for binary data. Since the SVD analysis is applicable to either quantitative or binary data, the dichotomization of quantitative data can be applied before or after the SVD. It should be noted that dichotomization before the matrix factorization could distort or enhance the signals in data matrix, which could, respectively, introduce the ambiguity or certainty of association structure. We conducted a small simulation, the results indicated that the two strategies appear to be compatible; however, a dichotomization before the matrix factorization performed poorly in sensitivity when the signal and non-signal were close.

SVD-Bin(δ) denotes the algorithm using SVD first to screen potential biclusters and then dichotomizing the data matrix to construct the biclusters by the edging algorithm with the threshold δ. Bin-SVD(δ) denotes the algorithm dichotomizing the data first and then applying SVD to identify potential biclusters followed by the edging algorithm to construct biclusters. Thus, the proposed two-step method leads to six algorithms: SVD-Bin(δ), Bin-SVD(δ), and NMF-Bin(*f*, δ), and Bin-NMF(*f*, δ) for quantitative data, and SVD(δ) and NMF(*f*, δ) for binary data. However, NMF is not applicable to matrices having rows or columns of all 0's, and no data analysis will be performed.

### Statistical Significance Test for Binary Biclusters

Koyutürk et al. [35] proposed a significance test for binary biclusters. If there is no association in the data matrix, each element can be assumed to be an outcome of independent Bernoulli trial with success probability *q*, which can be estimated by *k*/(*np*) where *k* is the number of 1's in the data matrix. The sum of the elements in an *n_r_*×*n_c_* submatrix follows a binominal distribution with parameters *n_r_n_c_* and *q*. The p-value of statistical significance test for an *n_r_*×*n_c_* bicluster is given by
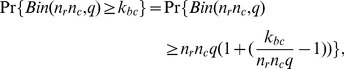
(6)where *k_bc_* (≥*n_r_n_c_*(1-δ)) is the number of the 1's in the bicluster. In general, it is difficult to numerically compute a very small p-value when *q* is small and/or *n_r_n_c_* is large. In these cases we used Chernoff's bound [38]

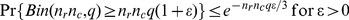
(7)to obtain the upper limit of the p-value from (6) which is
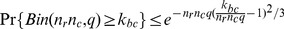
(8)if *k_bc_*>*n_r_n_c_q*. The inequality for *k_bc_* is usually true in bicluster analysis because only those biclusters which have more signals than the expected value are informative.

For each bicluster identified, a p-value or its upper bound was calculated. Since many biclusters would be identified, the Bonferroni correction [39] was used to control the overall type I error. The level of significance was set at 0.05/k, where k is the number of biclusters identified.

## Results

The two-step method was applied to two synthetic datasets and two real datasets. The synthetic datasets contained different overlapping and non-overlapping structures for illustrative purpose. The first real dataset was the *Saccharomyces cerevisiae* gene expression dataset [40] evaluated by the Bimax algorithm [16], and was used to demonstrate the difference between the proposed method and Bimax algorithm for a biclustering analysis of gene expression data. The data analyzed (analysis algorithms) in this paper are available in the R package *biclust*, which is extracted from *BicAT* toolbox [17]. The second real dataset was a *Salmonella* isolate dataset [41] genotyped by the Pulsed-Field Gel Electrophoresis with DNA band sizes representing the presence and absence of a feature in a location.

### Synthetic data

**Figure 3:**
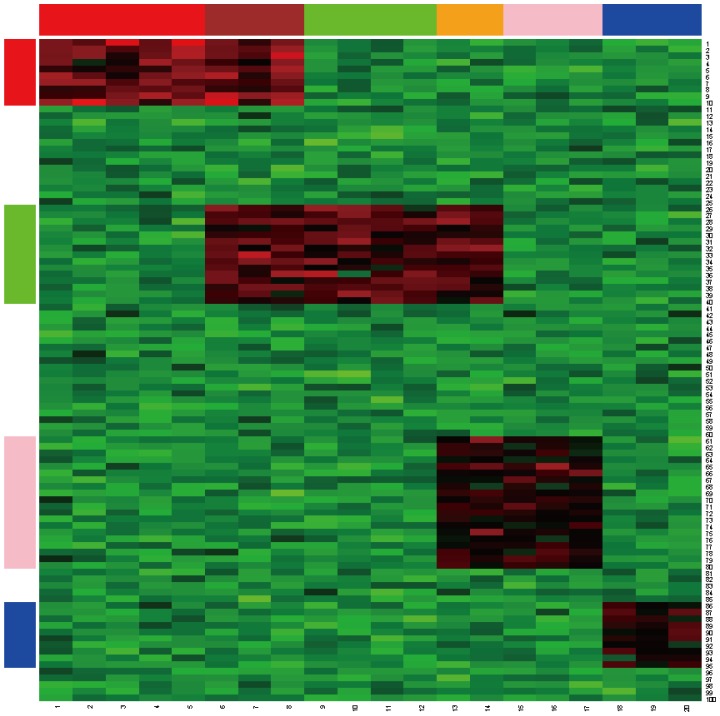
Heatmap of the original synthetic dataset biclustered in Figure 1. There are 4 biclusters colored as red, green, pink and blue in rows and columns; the brown and orange columns represent the overlapped columns of the biclusters.

**Figure 4:**
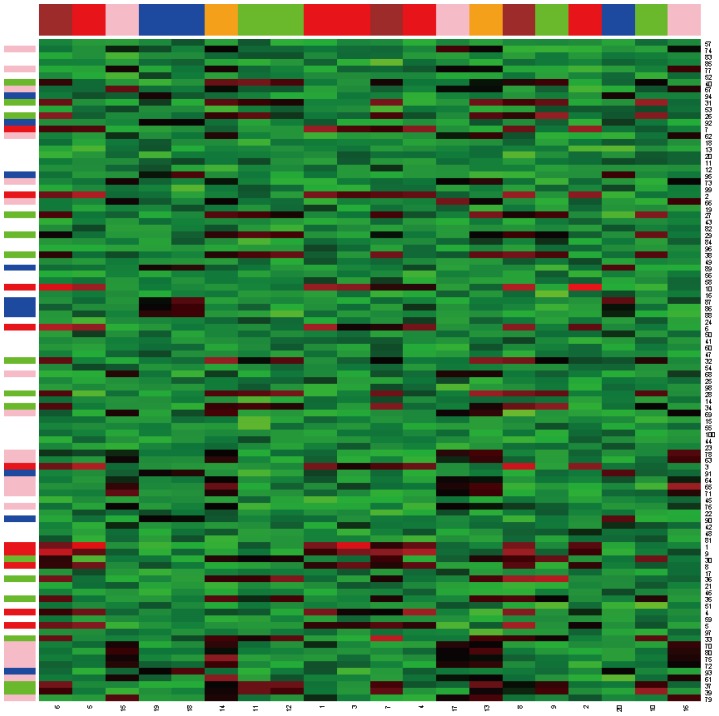
Heatmap of the randomly permuted synthetic data from Figure 3. This reflects the real data collected from an experiment.

Both synthetic datasets consisted of a data matrix of size 100×20. Four bicluster regions R1, R2, R3, and R4 with the sizes of 10×8, 15×9, 20×5, and 10×3, respectively, were considered. The first analysis considered quantitative data generated from normal distributions. The background data were generated from the normal random variable N(6, 0.8^2^). The four regions R1, R2, R3, and R4, were generated from the normal distribution N(12, 1.5^2^), N(11, 1.2^2^), N(11, 1^2^), and N(10, 1^2^), respectively. The first three biclusters, R1, R2, and R3, contained overlapping regions. R2 shares three columns with R1 and two columns with R3 (Figure 3). The four biclusters were colored as red, green, pink and blue in rows, and colored as red, brown, green, orange, pink and blue in columns. The brown and orange represented the overlapped columns of the biclusters. The simulated data were randomly permuted by column and row to ensure that the simulated data was more similar to real data. Figure 4 is the permutated data.

The Bin-NMF algorithm was not performed since there were rows and columns of all 0's. Note that deletion of those rows and columns to perform Bin-NMF could bias the results in the comparisons. The SVD-Bin(δ), Bin-SVD(δ), and NMF-Bin(*f*, δ) algorithms and several well known biclustering methods were applied to this dataset to compare their performance. The methods considered were Bimax [16], CC [18], xMotif [24], Spectral [21], Plaid [25], and FABIA [27]. The first five methods are available in the R package *biclust*, and FABIA is available at http://www.bioinf.jku.at/software/fabia/fabia. html. The default parameters were used in the analysis. In addition, the number of the biclusters *k* considered for Bimax(*k*), CC(*k*), xMotif(*k*), and FABIA(*k*) were from 4 to 8; these numbers were close to the true number of biclusters in order to have better performance for these biclustering methods. For Plaid, the number of biclusters was automatically determined in the algorithm. For Spectral, the number of principal components *f* was set from 3 to 5 using the independent and bistochastization rescaling algorithms. The two rescaling algorithms have a similar performance, only the results from the independent rescaling algorithm were reported, denoted as Spectral(*f*). The 2-means clustering algorithm was used to dichotomize the data for SVD-Bin(δ), Bin-SVD(δ), NMF-Bin(*f*, δ), Bimax and xMotif. The tolerance threshold δ for SVD and NMF was set at 0.3, 0.2 and 0.1.

One hundred simulated datasets were generated in the performance assessment, where each dataset was randomly generated from the same structure and distributions. For each simulated dataset the following performance measures were calculated: sensitivity (the proportion of correct identifications of signals in the bicluster regions out of the total number of signals), specificity (the proportion of the non-signals, which were not identified as signals, out of the total number of non-signals), perfect identification (all signals and all four biclusters were correctly identified), and the number of biclusters identified. Table 1 shows the results of performance measures which are the averages of 100 simulated datasets. The results show that NMF-Bin(4, δ), NMF-Bin(5, δ), Bin-SVD(δ) and SVD-Bin(δ) have the best overall performance. For these algorithms, δ = 0.2 slightly outperforms δ = 0.1 in sensitivity; however, δ = 0.1, which is a more stringent selection criterion than δ = 0.2, has better perfect identification and the number of clusters identified, as expected. When using same δ, Bin-SVD performs better than SVD-Bin in sensitivity but not in specificity. NMF-Bin performs better than SVD-Bin. Bimax and Spectral both have good specificity but poor sensitivity. CC and xMotif do not seem to perform well. Plaid does not require specifying the number of clusters and has similar performance behaviors as Bimax, good specificity and average sensitivity. For those published algorithms, FABIA appears to perform the best, as compared to 11 bicluster algorithms [27].

**Table 1:** Performance of SVD-Bin(δ), Bin-SVD(δ), NMF-Bin(*f*, δ), Bimax(*k*), CC(*k*), xMotif(*k*), Spectral(*f*), and FABIA(*k*) on a quantitative synthetic dataset where δ is the tolerance threshold, *f* is the number of factorization ranks, and *k* is the number of clusters.

	Sensitivity	Specificity	Proportion of perfect identification	Number of clusters
SVD-Bin(0.3)	0.961	0.984	0.02	6.67
SVD-Bin(0.2)	0.952	0.996	0.11	5.37
SVD-Bin(0.1)	0.915	1.000	0.25	4.41
Bin-SVD(0.3)	1.000	0.980	0	8.98
Bin-SVD(0.2)	0.999	0.994	0.03	7.08
Bin-SVD(0.1)	0.975	0.998	0.20	5.40
NMF-Bin(3,0.3)	0.874	0.996	0	3.67
NMF-Bin(4,0.3)	0.986	0.997	0.43	4.71
NMF-Bin(5,0.3)	0.988	0.995	0. 14	5.41
NMF-Bin(3,0.2)	0.871	0.999	0	3.53
NMF-Bin(4,0.2)	0.983	0.999	0.62	4.36
NMF-Bin(5,0.2)	0.978	0.998	0.29	5.1
NMF-Bin(3,0.1)	0.852	1.000	0	3.42
NMF-Bin(4,0.1)	0.960	1.000	0.58	4.13
NMF-Bin(5,0.1)	0.948	1.000	0.46	4.31
Bimax(4)	0.387	1.000	0	4
Bimax(5)	0.423	1.000	0	5
Bimax(6)	0.475	1.000	0	6
Bimax(7)	0.508	1.000	0	7
Bimax(8)	0.544	1.000	0	7.92
CC(4)	0.544	0.110	0	3.75
CC(5)	0.546	0.109	0	3.82
CC(6)	0.546	0.109	0	3.82
CC(7)	0.546	0.109	0	3.82
CC(8)	0.546	0.109	0	3.82
xMotif(4)	0.056	0.225	0	3.44
xMotif(5)	0.069	0.231	0	3.45
xMotif(6)	0.057	0.217	0	3.52
xMotif(7)	0.072	0.227	0	3.5
xMotif(8)	0.067	0.221	0	3.52
FABIA(4)	0.862	0.988	0.01	3.99
FABIA(5)	0.828	0.982	0.07	4.44
FABIA(6)	0.794	0.975	0.01	5.11
FABIA(7)	0.707	0.959	0.02	5.86
FABIA(8)	0.713	0.950	0	6.69
Spectral(3)	0.005	0.996	0	0.48
Spectral(4)	0.001	0.996	0	0.53
Spectral(5)	0.000	0.991	0	1.84
Plaid	0.508	0.913	0	4.76

The results are the average over 100 simulated datasets.

The second analysis considered binary data with the same four bicluster regions R1, R2, R3, and R4 in the data matrix. The background data was generated from a Bernoulli random variable with probability 0.95 for the non-signal and 0.05 for the signal; the bicluster regions were generated with probability 0.95 for the signal and 0.05 for the non-signals. Since the data were binary outcomes, only the SVD(δ) and Bimax algorithms were evaluated. Table 2 shows the results of performance measures from the averages of 100 simulated datasets. The results are consistent with the results in Table 1, SVD(δ) generally has much higher sensitivity than Bimax. Also, the binary data appear to have lower sensitivity and lower proportion of perfect identification than the corresponding estimates obtained from the quantitative data (Table 1).

**Table 2:** Performance of SVD(δ) and Bimax(*k*) on a binary synthetic dataset, where δ is the tolerance threshold and *k* is the number of clusters. The results are the average over 100 simulated datasets.

	Sensitivity	Specificity	Proportion of perfect identification	Number of clusters
SVD(0.3)	0.995	0.9669	0	10.13
SVD(0.2)	0.9599	0.9844	0.01	6.07
SVD(0.1)	0.8354	0.9967	0.02	4
Bimax(4)	0.2499	0.9972	0	4
Bimax(5)	0.2849	0.9964	0	5
Bimax(6)	0.3063	0.9959	0	6
Bimax(7)	0.3317	0.9952	0	7
Bimax(8)	0.3561	0.9946	0	8
Bimax(9)	0.3714	0.9942	0	9
Bimax(10)	0.3874	0.9938	0	10
Bimax(20)	0.5144	0.9898	0	20
Bimax(50)	0.7541	0.9801	0	50
Bimax(100)	0.8831	0.9698	0	95

The binary synthetic data can be considered as dichotomized quantitative data in which the biclusters have same proportions of signals and noises which will lead to 5% misclassification rates of the non-signals in background and signals in biclusters. This noise level is higher than the level in the quantitative synthetic data which have average misclassification rates 0.0075% in background and 3.21% in biclusters. We can find the influence of different noise levels by comparing results of Bin-SVD(δ) in Table 1 with the results of SVD(δ) in Table 2 because Bin-SVD(δ) after dichotomization is identical to SVD(δ). It shows that the performance for the quantitative (noisier) data in each evaluation metric is better than the binary synthetic data. In summary, the higher noise level will result in poor performance especially for small δ, and thus δ should not be too small when the data are noisy.

### *Saccharomyces cerevisiae* dataset

The *Saccharomyces cerevisiae* data set consisted of 419 probesets over 70 conditions (samples) available at R package *biclust* (http://cran.r-project.org/web/packages/biclust/index.html), and the data set was also analyzed in Bimax [16]. This dataset was analyzed to illustrate an approach to evaluate performance of dichotomized quantitative data. The NMF-Bin algorithm was not performed since this dataset consisted of negative gene expression level. Only the Bin-SVD, SVD-Bin, Bin-NMF, and Bimax algorithms were evaluated. For comparing with the Bimax algorithm, the data were dichotomized using the midrange which was used in Bimax. Accordingly, the data matrix was represented by a binary matrix with 1 and 0 representing the presence and absence of signals, respectively. Recall that a δ-biclustering algorithm aims at finding all maximal-dimensional submatrices in which the proportion of the non-signals is less than δ. Since the true biclusters of the data matrix were unknown, the performance was evaluated in terms of its ability to bicluster the observed signals in the data matrix. An ideal algorithm has high sensitivity, high specificity, and a small number of biclusters.

The biclustering results and processing times are summarized in Table 3. Bin-SVD(δ) and SVD-Bin(δ) appear to have similar performance for δ = 0.2 and 0.1. Bin-SVD(0.3) has much higher sensitivity together with a larger number of biclusters identified than Bin-SVD(0.3). Bin-NMF appears to perform much better than Bin-SVD and SVD-Bin for δ = 0.2 and 0.3. This analysis illustrates that δ can be used as a guidance criterion to identify sufficient number of biclusters. The sensitivity from the Bimax method was poor for this dataset. The analysis was performed for *k* = 5–200 (data not shown). It appears that increase the number of biclusters has little improvement in the sensitivity and the specificity stays at 1. Bimax appeared to identify many small perfect biclusters containing signals only. The fourth column shows the number of significance biclusters (p<0.05/k, where k is the number of biclusters identified), and SVD-Bin(0.3), Bin-SVD(0.3) and Bin-NMF(0.2) identify 6, 8,and 2 insignificant biclusters, which result from their small size with tolerated non-signals. The Bimax spent least processing time and the Bin-NMF consumed most time because the NMF is more time-consuming than the others.

**Table 3:** Performance of SVD-Bin(δ), Bin-SVD(δ), Bin-NMF(*f*, δ), and Bimax(*k*) on the *Saccharomyces cerevisiae* dataset where δ is the tolerance threshold, *f* is the number of factorization ranks, and *k* is the number of clusters.

	Sensitivity	Specificity	Number of clusters (k)	Number of significant clusters	Processing Time
SVD-Bin(0.3)	0.167	0.990	26	20	0.734
SVD-Bin(0.2)	0.082	0.997	14	14	0.657
SVD-Bin(0.1)	0.013	1.000	5	5	0.322
Bin-SVD(0.3)	0.410	0.957	56	48	1.825
Bin-SVD(0.2)	0.085	0.996	19	19	0.441
Bin-SVD(0.1)	0.015	1.000	4	4	0.228
Bin-NMF(70,0.3)*	0.653	0.955	55	55	8.443
Bin-NMF(70,0.2)*	0.269	0.991	21	19	8.466
Bin-NMF(70,0.1)*	No bicluster is found				5.073
Bimax(5)	0.027	1	5	5	0.007
Bimax(10)	0.036	1	10	10	0.007
Bimax(15)	0.083	1	15	15	0.010
Bimax(20)	0.084	1	20	20	0.008
Bimax(50)	0.094	1	50	50	0.009
Bimax(100)	0.168	1	100	100	0.011
Bimax(200)	0.175	1	200	200	0.011

* The number of the factors in NMF is the rank of the data matrix *f* = 70.

The biological interpretation of the biclusters identified can be evaluated using FuncAssociate (http://llama.mshri.on.ca/funcassociate_client/html/), GoTermFinder (http://www.yeastgenome.org/cgi-bin/GO/goTermFinder.pl), and/or Gene Ontology (http://www.geneontology.org/). An example of this evaluation can be found in [16], [20], [22], [27], [34].

### The *Salmonella* PFGE dataset

PFGE is a technique used for the separation of large DNA molecules by applying an electric field that periodically changes direction to a gel matrix. Standard methods for serotype identification of isolates are expensive and time-consuming [42], [43]. PFGE is currently considered as the “gold standard” technique used in subtyping of pathogenic bacteria. The *Salmonella* dataset consisted of 698 *Salmonella enterica* isolates collected from food producing animals, facilities and clinical diagnostic samples. The 698 isolates consisted of 4 serotypes: Heidelberg (n = 322), Javiana (n = 150), Newport(n = 91), and Typhimurium(n = 135). Each of the 698 isolates was characterized by the presence of about 15–20 distinct bands from a total of 71 bands of different sizes coded as 1 and 0, representing the presence and absence at the designated locations, respectively. Since the data were binary outcomes, only the SVD(δ), NMF(δ), and Bimax(*k*) algorithms were evaluated.

The PFGE data of 698 isolates consisted of four serotypes, whereas the samples in the synthetic and *Saccharomyces cerevisiae* datasets did not have group memberships. This PFGE dataset was evaluated in two aspects: overall performance and serotype-specific performance. The overall assessment did not consider the serotype information in the evaluation (Table 4); most of the biclusters identified are statistically significant except for SVD(0.3) which contains 3 insignificant biclusters. The results from SVD, NMF, and Bimax are consistent with the results in Tables 2 and 3. SVD and NMF have much higher sensitivity and slightly lower specificity than the Bimax method. Similar to the results from the *Saccharomyces cerevisiae* data, Bimax appeared to identify many small biclusters.

**Table 4:** Performance of SVD(δ), NMF(*f*, δ), and Bimax(*k*) on the PFGE dataset consisting of the 4 serotypes, Heidelberg (n = 322), Javiana (n = 150), Newport (n = 91), and Typhimurium (n = 135), for a total of 698 isolates with 71 bands.

	Sensitivity, based on observed data	Specificity, based on observed data	Number of clusters	Number of significant cluster	Processing time
SVD(0.3)	0.632	0.961	38	35	1.042
SVD(0.2)	0.439	0.980	14	14	0.379
SVD(0.1)	No bicluster is found				0.193
NMF(71, 0.3)*	0.425	0.997	19	19	16.833
NMF(71, 0.2)*	0.478	0.991	16	16	12.974
NMF(71, 0.1)*	No bicluster is found				15.496
Bimax(4)	0.029	1	4	4	0.009
Bimax(5)	0.029	1	5	5	0.009
Bimax(6)	0.038	1	6	6	0.009
Bimax(7)	0.040	1	7	7	0.009
Bimax(8)	0.040	1	8	8	0.008
Bimax(9)	0.043	1	9	9	0.008
Bimax(10)	0.048	1	10	10	0.008
Bimax(100)	0.104	1	100	100	0.014

* The number of the factors in NMF is the rank of the data matrix *f* = 71.

Tables 5 and 6 show the serotype-specific performance for SVD(0.2) and Bimax (100), respectively. The tables show the largest 10 biclusters identified and summarized by the serotype clusters. Ten largest biclusters in Table 5 included four Heidelberg and four Javiana biclusters, and one Newport and one Typhimurium bicluster. SVD(0.2) appeared to identify Heidelberg and Javiana reasonable well. In Table 6, the largest 10 out of the 100 biclusters included eight Typhimurium, and one Javiana and one Newport bicluster. Nine of the 10 biclusters shared two bands. A bicluster consisting of only two or three bands may not be useful to characterize a serotype. It is worth mentioning that the specificity in the overall assessment for Bimax(100) is 1 (Table 4); but the most of specificities are less than 1 in Table 6. This implied that those 100 biclusters identified consisted of signals of mixed serotypes.

**Table 5:** The ten largest biclusters out of 14 biclusters identified by SVD(0.2).

Majority	Sensitivity	Specificity	Isolates	Bands	log_10_p
Heidelberg	0.9845	0.8883	359	16	
cluster 1	0.9783	0.9814	322	12	≤−2402.38
cluster 2	0.9068	1	292	9	≤−1545.73
cluster 3	0.0807	0.9362	50	4	≤−106.22
cluster 4	0.0776	0.9707	36	5	≤−124.87
Javiana	0.6067	0.8595	168	8	
cluster 1	0.4267	0.8796	130	2	≤−146.11
cluster 2	0.1933	0.9818	39	3	≤−69.97
cluster 3	0.0733	0.9982	12	4	≤−42.18
cluster 4	0.02	0.9964	5	3	−6.75
Newport	0.0879	0.9951	11	3	
cluster 1	0.0879	0.9951	11	3	≤−12.37
Typhimurium	0.0296	0.9982	5	2	
cluster 1	0.0296	0.9982	5	2	−5.03

**Table 6:** The ten largest biclusters identified by Bimax(100).

Majority	Sensitivity	Specificity	Isolates	Bands	log_10_p
Typhimurium	0.8148	0.8686	184	5	
cluster 1	0.3801	0.9539	90	2	≤−166.96
cluster 2	0.3416	1	55	3	≤−153.05
cluster 3	0.4144	0.9942	78	2	≤−144.70
cluster 4	0.3908	0.9904	73	2	≤−135.42
cluster 5	0.2429	0.9315	72	2	≤−133.57
cluster 6	0.3026	0.9904	51	2	≤−94.61
cluster 7	0.2535	0.9773	48	2	≤−89.05
cluster 8	0.2482	0.9792	46	2	≤−85.34
Newport	0.2967	0.9489	58	2	
cluster 1	0.2328	0.9451	58	2	≤−107.60
Javiana	0.2	0.9672	48	2	
cluster 1	0.2482	0.9792	48	2	≤−89.05

The proposed method has better sensitivity and specificity for serotype identification than Bimax algorithm, and generally discovered more specific bands. In this method, both majority groups of the serotypes Heiderlberg and Javiana contain four biclusters, and each bicluster can be viewed as a subtype. The bands identified in each bicluster are overlapping. For example, there are 16 unique bands identified for the majority group of Heidelberg, but the total number of bands for the 4 clusters is 30. The commonly shared bands can be considered as the marker bands to distinguish Heidelberg from other serotypes. The non-overlapping bands exhibit the diversity among the PFGE patterns of Heidelberg isolates.

## Discussion

Bicluster analysis is specifically developed to identify which subsets of attributes are associated with which subsets of samples. A major difference between cluster analysis and bicluster analysis is that in cluster analysis each attribute or sample is assigned to one and only one cluster, while in bicluster analysis an attribute or sample can be part of more than one bicluster or of no bicluster.

In contrast with classification, bicluster analysis does not use the predefined class labels in identifying the local relationships between the attributes and samples. Each bicluster represents a specific sample-by-attribute relationship. Each set of samples corresponds to a set of sample specific attributes. The bicluster analysis is capable of providing an analysis for identifying subclasses or finding new classes (Table 3). In addition, bicluster analysis can be used as a preliminary analysis for determining the classes for supervised analysis.

Most variants of Biclustering are NP-complete problems [29] which require nondeterministic polynomial time for computation and whose faster algorithms are unknown, and most of the existing methods were developed using data mining strategies. Theoretical proofs of an algorithm could be infeasible. Evaluation and comparisons of biclustering algorithms are commonly based on synthetic data or/and real data which masked the class labels in bicluster analysis [10]–[14], [16], [22]–[24], [27]–[28]. Alternatively, some directly analyzed real data and evaluated the identified biclusters based on certain performance metrics [9], [18], [21], [26], or presented the analysis result [25].We used both synthetic datasets and real data to evaluate and compare the different algorithms.

Biclustering methods generally require specifying the number of biclusters *k* or the number of factorization ranks *f* (Table 1). The method, such as CC, requires pre-specifying the number of biclusters, identifies one bicluster at a time at the given threshold δ.The biclusters are successively extracted until the pre-specified number of clusters are identified, if the biclusters exist. This algorithm stops only when the pre-specified number of clusters has been identified. To cover a sufficient number of biclusters in the data matrix, the specified number is usually large. Also, some algorithms based on “masking” and random number of generation, the identified biclusters might not be reproducible and could reduce the identification of overlapping biclusters.

The matrix factorization methods, such as FABIA, and SVD- and NMF-based algorithms, extract quality biclusters from the candidate biclusters identified. As discussed, the existing published edging algorithms can fail to identify some biclusters occurred in the overlapped regions (Figure 1) on either row or column. The matrix factorization methods order the data matrix well because the extracted vectors aim to minimize the fitting errors orthogonally or non-orthogonally. We developed a novel edging method for the values of the ordered data but not the spectrums (eigenvectors). In the synthetic data analysis, it outperformed not only the spectral clustering but also the other existing methods when there are overlapping biclusters on either row or column.

As discussed, this approach needs a method to determine the boundary of the biclusters based on a tolerance constraint/assumption. Although the NMF method was shown to perform well in some cases in Table 1, this method also has the issue of reproducibility in that it can generate different sets of biclusters due to different initial conditions, and the biclusters identified earlier from the high rank components are not necessarily superior to those identified later. Carmona-Saez et al. [22] recommended an additional evaluation to select more stable and representative biclusters. In the SVD factorization, the principal components are ranked according to similarity data patterns. The biclusters extracted from high rank components are generally superior to those identified from lower rank components. Finally, the matrix factorization approach is based on the locality assumption which assumes that the projection planes can sufficiently reflect the local correlations structures. The insignificant components or low rank components may lead to small biclusters or no bicluster identified. The smaller submatrices might be filtered out either in Step 5 or having higher p-values. On the other hand, there could have some small biclusters that meet the δ and p-value criteria; thus, the proposed method averagely identified more biclusters than the true number of biclusters (see Table 1). An approach to identifying biclusters from those insignificant or low rank components is to incorporate a deletion step by removing the rows or columns with fewer signals so that the identified biclusters will have proportionally more signals. However, the deletion step may lead to over removal resulting smaller biclusters when applying to the significant or high rank components.”

Based on the analyses of the synthetic data and *Salmonella* example dataset, the proposed method is capable of identifying the overlapping biclusters, either on columns or on rows; and those overlapping rows or columns can be interpreted as the commonly regulating attributes or samples. In addition, the proposed method has better sensitivity and specificity for serotype identification than Bimax algorithm, and discovered more specific bands in the analysis of *Salmonella* PFGE fingerprints. The method will be helpful for the biologists to identify and understand characteristics of various *Salmonella* serotypes, and provides an approach to discovering new subtypes. The distinct band numbers and locations can be useful for serotype prediction and classification. The overlapping bands could be interpreted to simultaneously regulate the sub-serotypes.

The paper proposes a method to identify constant level biclusters. The method searches all candidate biclusters such that the proportion of contamination in the bicluster is less than a pre-specified threshold δ. The specification of δ is an important component in the proposed method, and we suggest that δ can be specified based on prior knowledge or observed experimental data. Alternatively, if the ratio (1-δ)/*q*, which measures how the biclusters contain larger proportion of signals than the whole data and should be larger than 1, is defined by the interest of the practitioners, the value of δ can be determined.

In summary, the proposed method is non-parametric and does not rely on assumptions of models or distributions such as [25], [28]. The proposed method is shown to be more effective than the commonly used methods for identifying constant level biclusters, and it also requires fewer input parameters which would be better for the users who do not have much prior knowledge in biclustering. Finally, the SVD and NMF algorithms used in this paper are the typical algorithms available in software R. The methods we have developed are applicable in any size data if the SVD, NMF and other matrix factorization methods work successfully. In this paper, we focuses on the performance (sensitivity, specificity, accuracy and statistical significance) of the bicluster identification, the efficiencies of speed and memory are not considered. Our procedures are available on request.
